# Nutritional management of the infant with chronic kidney disease stages 2–5 and on dialysis

**DOI:** 10.1007/s00467-022-05529-x

**Published:** 2022-04-05

**Authors:** Vanessa Shaw, Caroline Anderson, An Desloovere, Larry A. Greenbaum, Dieter Haffner, Christina L. Nelms, Fabio Paglialonga, Nonnie Polderman, Leila Qizalbash, José Renken-Terhaerdt, Stella Stabouli, Jetta Tuokkola, Johan Vande Walle, Bradley A. Warady, Rukshana Shroff

**Affiliations:** 1 University College London Great Ormond Street Hospital Institute of Child Health, 30 Guilford Street, London, WC1N 1EH UK; 2 University Hospital Southampton NHS Foundation Trust, Southampton, UK; 3 University Hospital Ghent, Ghent, Belgium; 4 Emory University, Atlanta, GA USA; 5 Children’s Hospital, Hannover Medical School, Hannover, Germany; 6 University of Nebraska, Kearney, NE USA; 7 Fondazione IRCCS Ca’ Granda Ospedale Maggiore Policlinico, Milan, Italy; 8 British Columbia Children’s Hospital, Vancouver, Canada; 9 Great North Children’s Hospital, Newcastle upon Tyne, UK; 10 Wilhelmina Children’s Hospital, University Medical Center Utrecht, Utrecht, The Netherlands; 11 1st Department of Pediatrics, Aristotle University Thessaloniki, Thessaloniki, Greece; 12 Children’s Hospital and Clinical Nutrition Unit, Internal Medicine and Rehabilitation, University of Helsinki and Helsinki University Hospital, Helsinki, Finland; 13 Children’s Mercy Kansas City, Kansas City, MO USA

**Keywords:** Infant, Nutrition, Growth, Energy, Protein, Phosphate, Chronic kidney disease

## Abstract

The nutritional management of children with chronic kidney disease (CKD) is of prime importance in meeting the challenge of maintaining normal growth and development in this population. The objective of this review is to integrate the Pediatric Renal Nutrition Taskforce clinical practice recommendations for children with CKD stages 2–5 and on dialysis, as they relate to the infant from full term birth up to 1 year of age, for healthcare professionals, including dietitians, physicians, and nurses. It addresses nutritional assessment, energy and protein requirements, delivery of the nutritional prescription, and necessary dietary modifications in the case of abnormal serum levels of calcium, phosphate, and potassium. We focus on the particular nutritional needs of infants with CKD for whom dietary recommendations for energy and protein, based on body weight, are higher compared with children over 1 year of age in order to support both linear and brain growth, which are normally maximal in the first 6 months of life. Attention to nutrition during infancy is important given that growth is predominantly nutrition dependent in the infantile phase and the growth of infants is acutely impaired by disruption to their nutritional intake, particularly during the first 6 months. Inadequate nutritional intake can result in the failure to achieve full adult height potential and an increased risk for abnormal neurodevelopment. We strongly suggest that physicians work closely with pediatric renal dietitians to ensure that the infant with CKD receives the best possible nutritional management to optimize their growth and development.

## The importance of nutrition in the growth of the infant

There are many reasons for poor growth in infants with chronic kidney disease (CKD) [1]. One of the most important of these is inadequate nutritional intake. Providing adequate nutrition is essential, particularly during the infantile phase of growth when growth is predominantly nutrition dependent. Inadequate nutritional intake may be due to anorexia, which is common in CKD, compounded by gastro-esophageal reflux (GER) with recurrent vomiting. The consequences of poor nutrition during infancy are suboptimal growth and impaired achievement of final height potential, abnormal body composition, developmental delay, a worsening of uremic symptoms such as vomiting, protein-energy wasting, and increased mortality. This period of nutritional vulnerability may be prolonged in CKD as the infantile phase of growth may extend from birth to 2–3 years of age [2]. Normally, linear growth slows towards the end of the infantile phase, followed by a transient acceleration of growth around 1 year of age at the beginning of the childhood phase of growth; the consequence of this delayed infantile phase in CKD is a slower overall rate of growth. In addition, many infants with CKD at birth, often due to congenital anomalies of the kidney and urinary tract, may be premature or small for gestational age (SGA) [3]. In a study of 101 infants with CKD stages 4–5 (CKD4–5), 26 were premature, 18 were SGA, while 10 were both premature and SGA; mean height standard deviation score (SDS) at birth was − 0.42 [4].

Optimizing the dietary prescription for the infant with CKD is of vital importance to promote optimal growth and reverse growth failure if present. While there are many factors associated with developmental delay and impaired cognition in this population, nutritional adequacy contributes to improved cognitive outcomes [5, 6]. Physicians should work closely with pediatric renal dietitians, with frequent dietetic contact, to ensure the infant with CKD receives the best possible nutritional management.

## Nutritional assessment

Nutritional assessment is the first step in determining the dietary prescription for the infant. A full description of the components of nutritional assessment has been published by the Pediatric Renal Nutrition Taskforce (PRNT) [7], including methods and tools for the assessment. The three key critical measurements for the infant are as follows: euvolemic (dry) weight, with adjustment of measured weight when indicated (e.g., when on dialysis or having nephrotic syndrome), length, and head circumference. These growth parameters need to be measured routinely in infants with a minimum recommended frequency of every 6 weeks throughout the first year of life. Weight measurements need to be undertaken more frequently at 2–4 weekly intervals if there are concerns about appropriate weight gain, or in advanced stages of CKD. Assessment of euvolemic body weight in infants on peritoneal dialysis (PD) is a particular challenge and weight gain resulting from fluid retention may be mistaken for acquisition of body mass. Indicators of excess weight due to fluid include edema on physical examination, and hypertension that is responsive to fluid removal during dialysis.

All measurements must be plotted serially on centile growth charts. The PRNT recommends using World Health Organization (WHO) growth charts, the worldwide standard for tracking growth in children younger than 2 years of age [8]. Weight-for-length (WFL) reflects body weight in proportion to length and should be monitored. Infants with low WFL may be underweight or malnourished; conversely, an infant with high WFL may be overweight or obese. WFL may also be plotted on WHO charts.

Growth is at its maximum in the first 12 weeks of life, with length increasing 1.2-fold and weight almost doubling in a healthy term infant compared to the time of birth [8]. Failure to gain weight is the most obvious signal for nutritional intervention as weight measurements are more sensitive markers of poor growth in infants in whom length measurements are difficult to perform and are often inaccurate. The longer an infant’s weight is static, the more difficult it is to regain their centile position on the growth chart: if weight is static for just 2 weeks in the first 3 months of life, there is a loss of 1 centile; if static for 4 weeks, 2 centiles are lost [9]. The normal rapid growth rate during the first year (being as high as 25 cm per year at birth, 18 cm per year at 6 months of age and slowing to 12 cm per year at 12 months of age) is even more difficult to regain when nutritional intake is poor and may lead to irreversible loss of final height potential. This is especially true when kidney replacement therapy is necessary during childhood, as this is associated with many impediments to catch-up growth, even with use of growth hormone therapy [10, 11]. Head circumference measurements are an additional tool and a small head circumference may indicate chronic inadequate nutrition, provided there are no other comorbidities that affect head circumference.

Disorder- or genetic condition-specific growth charts can be used for infants where growth potential may be altered, e.g., for those with trisomy 21 (Down syndrome), Wolf–Hirschhorn syndrome, and Prader–Willi syndrome [12–14]. Premature infants are also a special case: weight, length, and WFL for both gestational and chronological age should be plotted for the first year of life if born from 32 up to 37 weeks gestation, and through 2 years of age if born prior to 32 weeks gestation [15].

## Energy and protein requirements

The evidence and rationale for energy and protein requirements in infants have been described by the PRNT [9]. A novel term, the suggested dietary intake (SDI), has been adopted for dietary requirements, given the wide variations in the terms currently used. The lower and upper limits of the SDI for energy fall within the average daily amount of energy (i.e., the amount of energy considered sufficient to meet the needs of half the population) given in various international publications. The lower and upper limits of the SDI for protein fall within the average published amounts + 2 SD (i.e., the daily amount of protein considered sufficient to meet the needs of nearly all (97.5%) of the population) (Table 1). The SDI may be used for formulating the dietary prescription and assessing the adequacy of dietary intake for the individual infant.

**Table 1:** Suggested dietary intake (SDI) for energy and protein from birth at term to 12 months

Month	SDI energy (kcal/kg/day)	SDI protein (g/kg/day)	SDI protein (g/day)
0	93–107	1.52–2.5	8–12
1	93–120	1.52–1.8	8–12
2	93–120	1.4–1.52	8–12
3	82–98	1.4–1.52	8–12
4	82–98	1.3–1.52	9–13
5	72–82	1.3–1.52	9–13
6–9	72–82	1.1–1.3	9–14
10–11	72–82	1.1–1.3	9–15
12	72–120	0.9–1.14	11–14

Some 23 observational and retrospective studies, 18 of which included infants, reported the effects of specified energy intake measurements in children with CKD [8], most reporting that dietary energy intakes of around 100% of estimated energy requirements in those managed conservatively (i.e., not receiving dialysis) or on PD resulted in acceptable growth. Four trials studying resting energy expenditure or basal metabolic rate by indirect calorimetry in children showed no difference for those with CKD from healthy children after adjustment for lean body mass [16–19]. Consequently, the PRNT recommends that the initial prescription for energy intake should approximate that of the healthy infant. In those with suboptimal weight gain and linear growth, the energy intake should be adjusted towards the higher end of the SDI to promote optimal growth.

For infants receiving PD, the energy contribution from the dialysate glucose must be considered. Two studies have estimated energy contributions from dialysate in infants (7.5 ± 7 and 9.08 ± 4.13 kcal/kg/day, respectively) [20, 21], and it should be included in the calculation for total energy intake, recognizing the wide variation between patients and that the precise contribution depends on glucose concentration of the dialysate, time on dialysis, number of cycles and dwell times, and peritoneal membrane transporter status. Some infants may benefit from this additional source of energy if their dietary intake is insufficient; in contrast, dietary energy may need to be reduced if there is excessive weight gain.

Any restriction of dietary protein in the early stages of CKD should be avoided as a low protein intake may increase the risk of malnutrition, poor growth and protein-energy wasting. Inadequate protein not only impacts linear growth, but it also affects body composition, with a preponderance of fat laid down rather than lean tissue [22]. A randomized controlled trial in infants compared the effects of a low protein versus a normal protein diet over an average duration of 10 months to determine its potential impact on CKD progression [23]. At eight months of age, 24 infants (eGFR < 55 mL/min/1.73 m^2^) were randomized to receive either (what the authors considered to be) a low protein formula containing 1.4 ± 0.3 g/kg/day, with a protein-energy (PE) ratio (the percentage of total dietary energy derived from protein) of 5.6%; or control protein intake of 2.4 ± 0.4 g/kg/day, PE ratio of 10.4%. While the differences in protein intake did not have a significant impact on kidney function, the low protein diet group had significantly lower SDS for length and growth velocity compared with controls. To promote optimal growth, it is strongly recommended that dietary protein intake should be targeted at the upper end of the SDI. The ranges for protein SDI outlined in Table 1 are to be used for the initial prescription for infants. The protein intake at the lower end of the range is considered the minimum safe amount and should not be reduced below this level. Otherwise, it becomes the limiting factor for growth.

Due to the dialysate protein losses in infants on PD, protein intake may need to be higher than the SDI for infants managed conservatively. There are significant protein losses in the dialysate during PD, ranging from 0.28 g/kg/day in infants to 0.1 g/kg/day in adolescents [24]. Peritoneal transport characteristics and the increase of peritoneal protein losses that occur during peritonitis should also be considered when determining the individual dietary protein prescription. The recommendations for additional protein provided by the National Kidney Foundation Disease Outcomes Quality Initiative (KDOQI) in 2009 [25] are still valid: for infants on PD 0.15–0.3 g/kg/day; for infants on hemodialysis (HD) 0.1 g/kg/day.

In the case of an infant with persistently high blood urea levels, which may be the result of the infant feeding well and therefore having a protein intake higher than the upper end of the SDI range, the protein intake may be adjusted towards the lower end of the SDI, but not below this minimum safe level, after excluding other causes of high blood urea levels. A higher protein intake is not a problem per se if it does not result in unacceptably high urea levels, but it is important to recognize that higher protein intakes may negatively affect acid–base balance, and phosphate intake is also higher with a high protein intake, which may lead to hyperphosphatemia. In a study of 20 children on PD, dietary protein intake was negatively correlated not only with plasma bicarbonate, but also with total body bone mineral density, bone mineral content, and fat-free mass [26]. It is not expected that infants with CKD have urea levels in the normal range. High urea levels may indicate an excessive dietary protein relative to energy intake but may also be secondary to dehydration, steroid therapy or a catabolic state due to inadequate energy intake. In the latter case, firstly an increase in non-protein dietary energy may reduce urea levels. If this intervention is not sufficient then protein intake may be cautiously reduced, keeping in mind the minimum protein requirements for adequate nutrition and growth, while preserving energy intake. Infants with CKD stage 5 may require dialysis if dietary manipulation to reduce persistently high urea levels compromises their nutritional intake. Urea levels that are lower than expected for the stage of CKD may indicate insufficient dietary protein.

## Vitamins, minerals and trace elements

Little is known about the requirements for vitamins, minerals and trace elements in infants with CKD. Currently, the advice provided by KDOQI [25] would be reasonable to follow: at least 100% of the dietary reference intake for healthy children, with supplementation if dietary intake alone is insufficient; plus a water-soluble vitamin supplement for those with CKD stage 5 and on dialysis. An exception is vitamin A, as increased retinol levels have been reported in infants [27] and children with CKD and on maintenance dialysis, even without supplementation, with the associated development of hypercalcemia [28]. These high circulating levels of retinol are possibly a result of decreased glomerular filtration of the retinol–retinol-binding protein complex; reduced conversion of retinol to retinoic acid; and an accumulation of retinol-binding protein [29]. Vitamin D deficiency is common in CKD and evaluation, prevention and treatment of vitamin D deficiency is complex. Recommendations for the assessment of vitamin D status, optimal concentrations of 25-hydroxyvitamin D, and recommendations for native and active vitamin D supplementation are described elsewhere [30, 31]. Foods fortified with vitamin D may inadvertently lead to a cumulative excess of vitamin D, with consequent hypercalcemia [30]. These dairy products, margarines, and breakfast cereals do not feature largely in the infant’s diet; however, infant formulas are a significant source of vitamin D (around 1.5 µg/100 mL) and must be taken into account before prescribing vitamin D supplements.

## Feeding the infant

### Breastmilk and infant formula

Breastfeeding is the preferred method for feeding an infant with CKD, as recommended for the healthy population [32]. Infants with CKD2–5D may benefit from its low renal solute load, particularly with respect to its low phosphate and potassium content. Mothers who wish to breastfeed should be supported by all members of the medical and nursing teams. While many infants with CKD have been successfully breastfed for several months, some mothers can find it too stressful to breastfeed: they may be concerned that they do not know how much milk their baby is getting (and this may also be a negative view given by some healthcare professionals); or the sick baby may not latch onto the breast well. In such cases, reassurance must be given that the situation is being carefully monitored, and that supplementary feeds will be given as necessary. Breastfed infants need to feed every 2 to 3 h in order to obtain sufficient nutrition to maintain normal growth. If breastfeeding is not possible, the mother may wish to express her breast milk. If it becomes clear that the intake from breastfeeding alone is inadequate, or there is insufficient expressed breast milk (EBM), as demonstrated by a falling of the infant’s weight centile, then supplementary feeding should be provided with a whey-dominant infant formula (IF). If the mother does not wish to breastfeed at all, this type of IF can be given as the sole source of nutrition from birth. Whey-dominant IF has a protein and electrolyte content closer to that of breastmilk than casein-dominant IF, so is the preferred alternative and may be beneficial beyond the first year of life. Of course, whenever a mother wishes to continue breastfeeding, this should be prioritized and incorporated into the feeding plan, provided the infant’s nutritional intake is not compromised. Soya-based IF are not recommended under 12 months of age unless there is a specific medical indication, as there are concerns about their high phytoestrogen content.

Infants with CKD are prone to vomiting resulting from GER, delayed gastric emptying and gut dysmotility associated with decreased clearance of polypeptide hormones and cytokines [33, 34]. Additionally, for those on PD, vomiting may occur due to raised intra-abdominal pressure [35]. Normally, an IF feed volume of 150 mL/kg is sufficient to provide optimum nutrition. When growth is faltering, it is tempting to offer a higher volume of formula to improve nutritional intake. Unfortunately, this practice can be counterproductive as this higher volume often exacerbates vomiting and GER. Infants may actually benefit from a reduction in feed volume with an increase in the energy density of the feeds as described below.

Caution is needed with the polyuric infant who has a demand for fluids that is higher than normal. It would be unwise to attempt to meet all fluid requirements from IF as the infant will respond with increased vomiting. It is best to offer the necessary additional fluid as water between feeds, though some infants will also require additional electrolytes, principally sodium, due to high urinary or dialysate losses. The polyuric infant will readily drink water to maintain hydration.

### Concentrating feeds

When infants are reluctant to complete their feeds, or when there is a prescribed fluid restriction or a more energy and nutrient-dense feed is required to meet nutritional requirements, EBM and IF should be fortified.

Most standard IF are reconstituted to an approximate 13% concentration (i.e., 13 g powder to 100 mL water, providing 67 kcal and 1.3 g protein per 100 mL). The formula can be fortified to provide more nutrition in a given volume by concentrating up to 20% (i.e., 20 g powder to 100 mL water). Daily increases may be done in steps of 1–3%, depending on the infant’s tolerance of formula changes. Table 2 shows the effects of concentration on the formula profile. Infant formula powder may also be added to EBM at a concentration of 3–6% (i.e., 3–6 g infant formula powder to 100 mL EBM), increasing the total energy density up to 1 kcal/mL (Table 3). Breastmilk fortifiers may be used for preterm infants, contributing around 4 kcal/g, 0.26 g protein/g (follow manufacturer’s instructions for addition to breastmilk). They are designed to provide the increased requirements of the preterm infant for energy, protein, sodium, calcium, phosphate, vitamin D, and other nutrients. Their use must be carefully considered in the preterm infant with CKD.

**Table 2:** Concentrating whey-dominant infant formula (average composition per 100 mL)

Concentration	Energy (kcal)	Protein (g)	% CHO	% Fat	Na (mg)	K (mg)	Ca (mg)	PO_4_ (mg)
13 g powder/100 mL water (13%)	66	1.3	7.2	3.6	18	69	51	24
15 g powder/100 mL water (15%)	76	1.5	8.3	4.2	21	80	59	28
17 g powder/100 mL water (17%)	86	1.7	9.4	4.7	27	90	67	31
20 g powder/100 mL water (20%)	102	2.0	11.1	5.5	28	106	78	37

Effects of concentration on osmolality (mOsm/kg): 13% = 296; 15% = 339; 17% = 387; 20% = 451; ?upper limit = 500?

**Table 3:** Fortifying expressed breastmilk with whey-dominant infant formula (average composition per 100 mL)

Concentration	Energy (kcal)	Protein (g)	% CHO	% Fat	Na (mg)	K (mg)	Ca (mg)	PO_4_ (mg)
100 mL mature breastmilk	69	1.3	7.2	4.1	15	58	34	15
Plus 3 g infant formula powder	84	1.6	8.9	4.9	20	74	46	24
Plus 6 g infant formula powder	100	1.9	10.5	5.8	25	91	57	32

Breastmilk analysis from Public Health England: McCance and Widdowson’s *The Composition of Foods Integrated Dataset 2019* https://www.gov.uk/government/publications/composition-of-foods-integrated-dataset-cofid

The concentration of formula should be done in a gradual manner in order to maximize acceptance with respect to taste and gut tolerance since the resulting increase in osmolality [36] may cause diarrhea or exacerbate vomiting and GER. As the concentration of the formula is increased by the addition of more IF powder to a given volume of water, the renal solute load of the formula is also increased and may lead to excessive intakes of phosphate, potassium, and other minerals and vitamins, with toxic levels of vitamin A being of particular concern [28].

Alternatively, a patient-specific profile for energy and protein can be designed by using an IF at a normal concentration (or EBM if available) and adding powdered protein modules and/or energy modules (glucose polymers, fat emulsions, or combined carbohydrate and fat preparations). This method of fortification is preferable if concentrating the IF provides an intake of vitamin A, potassium, or phosphate beyond desirable levels. With this approach, it is essential to ensure that there is a sufficient intake of other vitamins, minerals, and trace elements. An example is given in Table 4. A simple recipe and caregiver education are required to reduce errors in formula preparation [37]. Commercially available energy and nutrient-dense liquid infant formulas, when available, may be useful as supplements to provide additional energy and protein.

**Table 4:** Modular approach to fortifying whey-dominant infant formula compared with concentrating formula (average composition per 100 mL)

Concentration	Energy (kcal)	Protein (g)	% CHO	% Fat	Na (mg)	K (mg)	Ca (mg)	PO_4_ (mg)
13 g infant formula powder (13%)	67	1.3	7.2	3.6	17	68	51	25
0.8 g protein powder	3	0.7	0	0	0*	0*	0*	0*
4 mL fat emulsion	18	0	0	2.0	0	0	0	0
4 g glucose polymer	15	0	3.8	0	0	0	0	0
per 100 mL	103	2.0	11.I	5.6	17	68	51	25
20 g infant formula powder (20%)	102	2.0	11.1	5.5	26	106	78	37

^*^Values vary according to brand of protein powder

To achieve optimal growth in the healthy infant with appropriate deposition of lean and fat tissue, the PE ratio of the infant formula should ideally be between 7 and 12%; a high PE ratio is required for accelerated weight gain or catch-up growth. The WHO suggests that a rate of weight gain of 10 g/kg/day requires a daily intake of 126 kcal/kg, 2.8 g protein/kg, and a PE ratio of 8.9% [22]. In infants with CKD, the addition of non-protein energy sources (carbohydrate and fat) to a formula can be used to control elevated blood urea or potassium levels resulting from poor energy intake, catabolism, and consequent tissue breakdown, which lowers the PE ratio. In these situations, it is important to ensure that at least the SDI for protein is provided. A PE ratio of 5.3–6.4% has been shown to support weight gain and linear growth in children aged 0–2 years, provided at least 100% of protein requirements were given [38]; the lower PE ratio reflected an increase in energy provision rather than a low protein intake.

The impact of adding carbohydrate and fat supplements on the osmolality of IF is largely unknown, though one study has shown the addition of 2 g glucose polymer powder per 100 mL IF increased the osmolality by 31 mOsm/kg [36]. It is recommended that carbohydrate modules are added in increments, e.g., 1% daily (1 g added to 100 mL formula or EBM per day, yielding an additional 4 kcal/100 mL). Such gradual increase allows for the concentration at which the infant becomes intolerant, as reflected by the development of loose stools/increased vomiting, to be identified. If glucose polymers are not available, then glucose or sucrose may be used, but as the osmotic effect of mono- and disaccharides on the gut is greater than a polymer, less can be used per given volume of formula. Also, the intense sweetness of mono- and disaccharides may limit their use. The dental hygiene of the infant’s emerging teeth must also be considered. Tolerance to carbohydrate concentration of the feed depends on the type of supplement; the age of the infant; the maturity and absorptive capacity of the gut, with some infants more tolerant of a more rapid addition of carbohydrate than others (Table 5).

**Table 5:** Energy modules added to whey-dominant infant formula

Energy module	Age	Amount of CHO/fat module added to formula	Final concentration CHO/fat in formula (% or g/100 mL)
Glucose polymer	< 6 months	3–5 g (+ 7 g CHO from infant formula)	10–12
	6 months–1 year	5–8 g (+ 7 g CHO from infant formula)	12–15
Glucose	< 1 year	1–2 g (+ 7 g CHO from infant formula)	Maximum 8–9
Sucrose	< 1 year	2–3 g (+ 7 g CHO from infant formula)	Maximum 9–10
Fat emulsion (50% fat content)	< 1 year	3–5 mL (+ 3.5 g fat from infant formula)	5–6

The fat source of commercial fat emulsions is polyunsaturated oils with a favorable omega-3 to omega-6 fatty acid ratio. They should also be added incrementally, e.g., 1% daily (1 mL added to 100 mL formula or EBM per day) to provide an increase of 0.5 g fat per 100 mL (an additional 5 kcal/100 mL). The increased fat content may delay gastric emptying and cause nausea and vomiting. If emulsions are not available, then oils may be used, but little is known about the type and amount that can be added to IF. Oils will have little effect on osmolality, but will settle out on standing. A bottle of IF fortified with oil must be shaken frequently throughout the feeding period to ensure that the oil is dispersed and, therefore, consumed.

Powdered protein modules are added to formulas or EBM to provide a specific amount of protein per kg of body weight. If they are used in infants, for instance to compensate for protein losses in dialysate, they should be added in small increments of 0.1 g protein/kg/day, with urea levels serially monitored to detect excessive intake. Some protein modules may contain appreciable amounts of phosphate so serum phosphate levels may be impacted if large amounts are used.

### Complementary feeding

Exclusive breastfeeding for the first six months of life, is recommended by WHO [32], and solid foods should be introduced to the infant with CKD as for healthy infants. However, the age when solids are introduced should be managed individually. Delayed exposure to pureed and more textured foods may cause a delay in the development of oral motor skills and result in feeding problems [39]. The nutritional content of any solid foods taken must be balanced against that provided by the formula in order to maintain an optimum intake of energy, protein and other nutrients during the weaning period. A healthy balanced diet (adapted to any phosphate or potassium restrictions that may be required) with a wide range of food choices should be offered, with a variety of textures according to the infant’s cues and oral motor skills. However, the infant with CKD may be reluctant to take an oral diet for a number of reasons. Acidosis, anemia and uremia negatively influence oral intake if not corrected. Appetite for both feeds and solids may be also be impacted by persistent GER and gut dysmotility. It seems likely that the reduced smell and taste sensations recognized in older children with CKD [40], which worsens as CKD progresses, also affect the infant’s appetite. The polyuric infant preferentially drinks large amounts of water, which also reduces appetite. Finally, the many medications that may be required further affect what the infant is prepared to have in its mouth and swallow. The combination of these elements can lead to food aversion that may not resolve until normal kidney function is restored with a transplant.

## Tackling poor growth

The PRNT recommends prompt intervention once there is a deterioration in the infant’s weight centile. The first step is to address any correctable medical causes of reduced dietary intake and optimizing dialysis. GER may be treated with alginates, antacids, histamine H_2_ receptor antagonists, proton pump inhibitors, and prokinetics. Sucralfate, although an effective treatment for GER, should not be used in infants with CKD since it contains aluminum hydroxide that may lead to aluminum intoxication in kidney failure, which has been associated with bone and neurological disease. Infants with renal dysplasia may have significant urinary losses of salt and water and become both salt and water depleted. Sodium is an important growth factor and salt deprivation inhibits growth, even if the intake of energy and other nutrients is sufficient. This failure to gain weight can be reversed with sodium and water supplementation. Height SDS was shown to improve by 1.83 SD in 24 polyuric infants (92% with obstructive uropathy and dysplasia) over a 2-year period when formula was supplemented with up to 4 mmol/100 mL of sodium chloride and/or sodium bicarbonate [41]. Correction of acidosis with base supplementation is also necessary. Acidosis is common in all causes of CKD when GFR falls below 25 mL/min/1.73 m^2^, and even moderate acidosis is associated with poor growth due to negative effects on bone metabolism and muscle synthesis. KDOQI recommends maintaining serum bicarbonate at 22 mmol/L or higher [25]. Inadequate PD may be an important factor for undernutrition and poor growth, and in certain cases, a switch to intensified, often daily, HD may be indicated.

If poor growth persists, even when the above issues have been addressed, this is most likely due to inadequate nutritional intake. In this case, nutritional supplementation should be started.

### Oral nutritional supplementation

To improve nutritional intake, EBM can be supplemented by the addition of IF powder; IF can be concentrated or fortified with energy and protein modules (Tables 2–4). Foods with high nutritional value should be offered, ensuring the appropriate texture for infants. High biological value protein foods such as meat, poultry, fish, eggs, and milk products, e.g., cheese, yogurt, fromage frais, provide the right proportion of essential amino acids for growth and protein repletion. Low biological value protein foods from plant sources have an incomplete profile of essential amino acids: pulses (peas, beans, lentils); ground nuts or nut butters; cereals (grains) such as rice, pasta, couscous; potatoes and other starchy roots and tubers; fruits and vegetables. However, these foods may need to be given preferentially if there is a need for reduced protein intake in the case of rising blood urea levels. The continued provision of breastmilk and IF will ensure a balance of amino acids to maintain growth. The amount of some of these foods may need to be limited due to their phosphate and potassium content. Savory foods can be fortified with energy by adding vegetable margarines or oils, preferably those high in omega-3 fats, e.g., soya, walnut, linseed, rapeseed, or high in monounsaturated fat, e.g. olive oil. Sugar, jams, syrups and glucose can be added to sweet foods. Energy modules can also be added to sweet and savory foods. Glucose polymers can be added to water, starting with 5% (5 g added to 100 mL) and increasing to 10% for the younger infant and 15% for the older infant as tolerated and needed. Sugar and glucose may also be used, but the quantity may be limited due to their sweet taste; these infants prefer salty, savory flavors over sweetness.

### Enteral tube feeding

Infants who are unable to meet their nutritional requirements orally should be started on supplemental or exclusive enteral tube feeding in order to improve their nutritional status. The PRNT suggests that there should be prompt intervention once a deterioration in weight centile is noted. Intervention may be appropriate at any age but is especially important in infants, where it is likely to reverse poor linear growth. Eight retrospective studies, in which tube feeds were either supplemental to oral intake or provided up to 100% of energy and protein requirements, showed improvement in weight and body mass index SDS in children < 2 years [4, 38, 42–47]. Six of these studies also showed an improvement in height SDS [4, 38, 42, 44, 45, 47].

A nasogastric (NG) tube is the preferred option for short-term enteral feeding (EF), while a gastrostomy device (GD) is preferable for long-term EF. An NG tube may be considered as a bridging option to a long-term GD, improving the nutritional status of the infant in preparation for placement of the GD. The healthcare team must involve the infant’s parents/caregivers in the decision to tube feed, explaining why it is necessary, the process of tube feeding, the different types of tubes and devices available, and emphasizing the possible benefits, including a reduction in parental anxiety around oral feeding; using the tube for the administration of medications and any additional fluid that might be needed; improving growth, and thus reducing the time to reach the desired size for a kidney transplant. In addition, complications with the use of EF tubes must be explained, especially the risk of aspiration if there is a displacement of the NG tube into the airway.

Preparations and investigations prior to insertion of a GD and techniques used for their insertion are fully described elsewhere by the PRNT [48]. Table 6 summarizes recommendations for placement of a GD in the child who is receiving PD. There is no evidence for when it is safe to start using a GD post-insertion in infants with CKD or on dialysis; the European Society for Pediatric Gastroenterology, Hepatology and Nutrition (ESPGHAN) suggests 4–6 h post-insertion for the general pediatric population [49]. The PRNT suggests starting with water or other appropriate clear fluid 2 h post-insertion; 30 mL would be appropriate for an infant. If this is tolerated, then at 3 h, 50% of the usual feed volume rate can be given as water. At 6 h, the formula or EBM can be given instead of water and the amount gradually increased until the full prescription is achieved.

**Table 6:** Summary of PRNT recommendations for placement of gastrostomy devices while on PD [45]

In a child who is likely to need PD, and in whom enteral tube feeding is required, gastrostomy tube insertion by PEG or RIG should, whenever possible, be performed before placement of a PD catheter
A gastrostomy device can be inserted simultaneously with a PD catheter if the gastrostomy is placed by open surgery or PLAG
In a child who is already receiving PD an open or laparoscopically assisted gastrostomy is the preferred procedure
Antibiotic prophylaxis, based on local antibiotic sensitivities, is recommended for all children undergoing gastrostomy placement
Children who are already established on PD or who receive a gastrostomy at the same time as a PD catheter should receive broad spectrum antibiotic and antifungal prophylaxis in the peri-operative period of gastrostomy placement
PD should be withheld for 24 h or longer after gastrostomy placement if it is clinically safe to do so

*PD* peritoneal dialysis, *PEG* percutaneous endoscopic gastrostomy, *PLAG* percutaneous laparoscopic assisted gastrostomy, *RIG* percutaneous radiologically inserted gastrostomy

Tube feeding may be exclusive or supplementary to oral feeding. Gravity bolus feeds can be given either as supplementary feeds after meals or to complete unfinished oral feeds, or all the infant’s nutrition can be given as boluses. If EF pumps are available, overnight continuous feeds can be given through the GD. This takes away the anxiety of parents/caregivers in their battle to feed an infant who is reluctant or refuses to feed by mouth. Continuous NG overnight feeding at home poses a significant risk for aspiration of the formula/EBM if used unsupervised, which can be fatal. A local risk assessment should be conducted.

Infant formulas and EBM are preferred for EF and can be supplemented, if necessary, as described above. If caregivers wish to prepare their infant’s tube feeds by blending foods, this should be done under the supervision of a qualified dietitian who will advise about safety issues with this practice, including nutritional quality, microbial contamination and appropriate equipment and administration [50]. The use of commercial baby foods would be preferable as they are sterile and have a known nutritional profile.

Although EF can ensure that an infant is getting their complete nutritional prescription, it is often associated with long-lasting feeding difficulties: chewing and swallowing problems, and food refusal [51]; and poor development of oral motor skills [39]. Ongoing oral stimulation is essential in infants who have prolonged tube feeding to help them to transition to normal feeding once they have had a successful transplant [52, 53]. Even when an infant is dependent on tube feeds, caregivers can encourage continued oral intake by offering drinks from a bottle or cup and pureed or more textured foods when developmentally ready; engaging in messy play and joining family mealtimes, even if nothing is eaten; giving positive oral experiences, such as touching and kissing the mouth area; giving non-nutritive oral stimulation such as a pacifier to suck, mouthing toys; encouraging self-feeding, licking, and tasting of foods with no pressure to swallow. Support from a speech and language therapist, psychologist, or family therapist can be helpful when there are significant feeding difficulties [39].

If growth continues to be poor, despite the provision of adequate nutrition through supplementation of the oral diet or enteral tube feeding, growth hormone therapy may be considered in the infant to enhance growth [11].

### Management of vomiting

Vomiting can be an ongoing problem for some infants, even in those who are receiving tube feeding. This persistent vomiting becomes very stressful for families as well as having a negative impact on oral feeding for the infant. Having exhausted the medical therapies outlined above, some strategies that may help are as follows: give smaller, more frequent bolus feeds; concentrate the formula to allow a reduction in the feed volume; slow delivery of the feed by continuous infusion via EF pump [39]; reduce the rate of delivery and give the feed over a more extended period of time. Continuous feeding may lead to higher pH values in the stomach which can promote bacterial growth, particularly affecting those with significant dysmotility [49]. It is therefore recommended that there is sufficient time off the EF pump to allow the stomach pH to return to normal.

If growth is negatively affected by persistent vomiting, upper gastrointestinal contrast study and a pH study should be performed to exclude malrotation and to define the severity of GER. Post-pyloric, or trans-gastric, feeding via nasojejunal or gastrojejunostomy may be considered, but ESPGHAN recommends this route of feeding only if there is severe GER disease, gastroparesis or gastric outflow obstruction [49] because of frequent complications. Nasojejunal tubes are easily displaced and migrate up into the stomach; gastrojejunal tubes or jejunostomy are the preferred alternatives. For both gastrojejunal and nasojejunal tubes, formula must be delivered by continuous infusion, as boluses entering the jejunum are poorly tolerated. In a study of 101 infants diagnosed with CKD in the first 6 months of life, 13 required a Nissen fundoplication for persistent vomiting [4].

## Dietary modifications

The PRNT has published a full account of the nutritional management of calcium and phosphate [54] and potassium [55]. While dietary intakes may need to be adjusted to maintain serum levels within the age-appropriate normal range, restrictions should be limited as much as possible so as not to worsen any pre-existing reluctance to eat. Any changes in nutritional management should be based on trends of serial results rather than a single result and without compromising the infant’s overall nutritional status.

### Calcium

Calcium is necessary for bone mineralization and to prevent fractures [56]; therefore, a low dietary calcium intake is of concern. Conversely, a high calcium intake may lead to vascular calcification, progressive vessel stiffness and left ventricular failure [57–61]. Calcium intake in the infant is unlikely to be excessive or deficient if nutritional requirements are met from breastmilk, IF, and some contribution from solid foods. Calcium intake should provide at least 100% of the SDI (Table 7) but be no more than twice the SDI. In special circumstances, such as for infants with mineral-depleted bone, a higher calcium intake of > 200% SDI may be needed to replenish bone calcium stores and is likely to require calcium supplementation in addition to dietary calcium. In the case of persistent hypocalcemia or a high parathyroid hormone level, calcium intake should be increased above 200% SDI for a short period under close medical supervision [54]. Additional calcium can be provided through calcium supplementation and high calcium dialysate. The use of calcium-rich foods, such as dairy products (cheese, yogurt, fromage frais) which infants are most willing to eat, may need to be limited as these will also increase dietary phosphate intake. Absorption of calcium requires adequate vitamin D and often requires supplementation (usually both native and active forms) [30, 31]. It should be noted that frequent vomiting may lead to metabolic alkalosis, which in turn might affect the interpretation of serum calcium levels—ionized calcium levels must be measured so that hypocalcemia can be detected and managed appropriately.

**Table 7:** Suggested dietary intake (SDI) for calcium and phosphate

Age (years)	SDI calcium (mg/day)	SDI phosphate (mg/day)
0 to < 4 months	220	120
4 to < 12 months	330–540	275–420

In the case of persistent mild to moderate hypercalcemia, management is mainly medical: reducing or stopping calcium supplements, calcium-based phosphate binders, and native and active vitamin D, and using lower calcium dialysate. A temporary reduction of dietary calcium may also be necessary. If hypercalcemia is severe and persists, a nutritionally complete specialized low calcium formula may fully or partially replace IF or breastmilk (mothers should be supported to express and safely store their breastmilk until it can be fed to their infant). In addition to dairy products, other calcium-rich foods may need to be restricted: green-leafy vegetables, beans, ground nuts and nut butters, and calcium-fortified foods. Using deionized or distilled water to reconstitute formula also reduces calcium intake. Regular assessment of dietary calcium is required, especially when calcium intake is reduced below the SDI.

### Phosphate

Table 7 shows the SDI for phosphate. Serum phosphate is likely to be within the normal range if the infant is fed breastmilk or whey-dominant IF. However, phosphate retention begins early in the course of CKD, and if there is either persistent hyperphosphatemia or persistent hyperparathyroidism in the presence of normal calcium levels, dietary phosphate should not exceed the SDI for young infants, and intake should be reduced to the lower end of the SDI range for infants older than 6 months [54]. The introduction of complementary solid foods that are easy to swallow, particularly soft dairy foods and eggs, increases phosphate intake. Whole grain cereals, although higher in phosphate than refined varieties, need not be restricted as the inclusion of fiber in the diet is beneficial. Foods with a suitably lower phosphate content are shown in Table 8. Processed foods containing phosphate additives should be avoided as they are a source of highly bioavailable inorganic phosphate, which is almost completely absorbed [62].

**Table 8:** Controlling phosphate and potassium intakes

		Food type		
Comment	Lower phosphate options		Lower potassium options	Comment
Continue beyond 1 year	Breastmilk, whey-dominant infant formula	Milk	Breastmilk, whey-dominant infant formula	Continue beyond 1 year
Portion size may need to be controlled	Cottage, cream, ricotta cheese; paneer	Cheese, yogurt	Any type	Portion size may need to be controlled
	Baby cereals; rice, pasta, noodles, couscous, millet, bulgur wheat, semolina, tapioca; bread, chapattis; potatoes	Starchy foods	Baby cereals; rice, pasta, noodles, couscous, millet, bulgur wheat, semolina, tapioca; bread, chapattis High K: potatoes—cut into small pieces and double boil, discard cooking water	
Portion size may need to be controlled	Fresh, unprocessed, any type	Meat, chicken, fish	Fresh, unprocessed, any type	Portion size may need to be controlled
	Egg white	Eggs	Whole egg	
	Any type, e.g., lentils, split peas, chickpeas; black eye, broad, butter, cannellini, red kidney beans; tofu; houmous	Pulses, legumes	Lentils, split peas, chickpeas; cannellini, red kidney beans; tofu; houmous High K: black eye, broad, butter beans	Portion size may need to be controlled
Portion size may need to be controlled	Any type, e.g., almonds, hazelnuts, peanuts, walnuts; pumpkin, sesame, sunflower seeds; tahini paste	Nuts and seeds (ground, pastes, butters)	Almonds, hazelnuts, peanuts, walnuts; sesame and sunflower seeds; tahini paste High K: pumpkin seeds	Portion size may need to be controlled
	Any type, e.g., avocado, broccoli, brussels sprouts, butternut squash, cabbage, carrot, cauliflower, courgette, okra, plantain, pumpkin, swede, sweet potato, tomato, yam	Vegetables	Broccoli, butternut squash, cabbage, carrot, cauliflower, courgette, pumpkin, swede High K: avocado, brussels sprouts, okra, plantain, sweet potato, tomato, yam	Portion size may need to be controlled
	Any type, e.g., apple, apricot, banana, blueberries, lychees, mango, melon, orange, papaya, pear, pineapple, raspberries, strawberries	Fruit	Apple, blueberries, lychees, pear, pineapple, raspberries High K: apricot, banana, mango, melon, orange, papaya, strawberries	Portion size may need to be controlled
Avoid	Phosphate additives—bioavailability up to 100%	Food additives	Potassium additives—bioavailability 90–100%	Avoid

Although breastmilk and whey-dominant IF are naturally low in phosphate (15 mg/100 mL and average 30 mg/100 mL, respectively), a specialized renal-specific low potassium infant formula, that also has a lower phosphate content, may be given so that dietary restrictions can be liberalized, allowing a greater variety of food choices, which may encourage more normal development and behaviors around food. Table 9 shows the composition of a typical renal-specific formula and how to adapt a whey-based IF to reduce phosphate content. Diluting the IF needs to be done with extreme caution as this also reduces the energy, protein, vitamin, and mineral content of the formula. Energy and protein modules must be added, together with a suitable vitamin and mineral preparation, to restore the full composition and maintain nutritional adequacy.

**Table 9:** Specialized and adapted infant formulas per 100 mL

	Energy (kcal)	Protein (g)	CHO (g)	Fat (g)	Na (mg)	K (mg)	Ca (mg)	PO_4_ (mg)
Typical whey-dominant infant formula								
3 scoops powder (13 g) per 100 mL	66	1.3	7.2	3.6	17	68	51	25
Typical low calcium infant formula								
13 g powder per 100 mL	66	1.9	7.0	3.4	29	84	< 7	46
Typical renal-specific low potassium infant formula								
13 g powder per 100 mL	65	1.0	8.2	3.1	31	14	16	12
Diluted whey-dominant infant formula providing less calcium, phosphate and potassium								
2 scoops powder (9 g)	47	0.9	5.0	2.5	12	47	35	17
0.5 g protein powder	2	0.5	0	0	0*	0*	0*	0*
2.5 mL fat emulsion	11	0	0	1.3	0	0	7	0
2 g glucose polymer	8	0	2.0	0	0	0	0	0
Per 100 mL	68	1.4	7.0	3.8	12	47	42	17

A vitamin supplement (without vitamin A) and mineral supplement may be necessary to achieve nutritional adequacy. Calcium content may increase depending on calcium content of local water supply. The figures here assume using deionized or distilled water for reconstitution (check calcium content of any mineral supplement)

^*^Values vary according to brand of protein powder

Renal-specific low potassium infant formulas should not be used as the sole source of nutrition as their low potassium content may cause a precipitous fall in serum potassium. Finally, phosphate binders may be required with more advanced CKD and the development of hyperphosphatemia (added to bottle feeds and moist foods), as any dietary restriction may have a negative impact on an existing reluctance to eat and further decrease intake of solids.

In the case of hypophosphatemia, dietary phosphate should be increased so as to achieve age-appropriate serum phosphate levels. The easiest way to do this is to offer more milk- and egg-based foods; phosphate supplements may be necessary if this approach is not tolerated or does not correct the hypophosphatemia.

### Potassium

Infants are at risk for developing hyperkalemia in association with moderate to severe CKD. Whereas a reduced potassium intake is usually indicated, hyperkalemia can result from catabolism when there is deficient energy intake [9]. This can be resolved by the addition of energy modules to the infant’s usual formula or EBM (Table 5). There are no data to suggest potassium requirements for children with CKD. Extrapolating from adult data, KDOQI recommends an initial target potassium intake of 1–3 mmol/kg/day for infants to maintain a normal serum concentration [25]; however, the PRNT recommends that dietary potassium intake is only adjusted if the serum potassium level is outside the normal range: 3.5–5.0 mmol/L in infants, and 3.5–5.5 mmol/L in neonates, based on serial measurements [55].

Before adjusting the diet, the many non-dietary causes of hyperkalemia, such as pseudohyperkalemia, medications that affect serum potassium (e.g., some laxatives, angiotensin-converting enzyme inhibitors, renin–angiotensin–aldosterone system inhibitors, and beta-blockers), metabolic acidosis, constipation, and the dialysis prescription, should be addressed [55]. Severe, life-threatening hyperkalemia requires rapid medical intervention with discontinuation of all sources of potassium from medications, parenteral fluids, formulas, and food.

Serum potassium is likely to be within the normal range in the infant with mild to moderate CKD if fed breastmilk or whey-dominant IF. However, if there are persistent episodes of hyperkalemia, reduced intake of potassium can be achieved by gradually replacing some of the breastmilk or IF with a specialized renal-specific low potassium IF (Table 9). Serum potassium levels need to be regularly checked to ensure they are maintained in the normal range. Attention also needs to be paid to the changes in nutrient profile, as there is often decreased calcium and phosphate content and, for some products, an increased sodium content due to the renal-specific formula. If a renal-specific low potassium IF is not available, a whey-based IF can be adapted to reduce its potassium content (Table 9). As described above, diluting an IF needs to be done with great caution as this also reduces the energy, protein, vitamin, and mineral content. If EBM is diluted, it too must have nutritional adequacy restored by the addition of energy and protein modules and an appropriate vitamin and mineral supplement given. There is no evidence that reducing maternal dietary potassium has any impact on the potassium content of breastmilk. Generally, a renal-specific low potassium IF should only be used as the sole source of nutrition in the short term (hours rather than days) as the low potassium content may cause a rapid fall in serum potassium. If used solely in the initial treatment of moderate to severe hyperkalemia, whey-dominant IF or breastmilk should be introduced as soon as serum potassium levels allow. Some infants may benefit from the extended use of a renal-specific formula; however, caution is advised as in addition to a decreased potassium intake, there will be decreased calcium and phosphate intakes and, with some formulas, an increased sodium intake. An adult renal-specific low potassium formula can also be used in infants to reduce serum potassium [63], but the nutritional profile needs to be carefully checked for its suitability.

An alternative therapeutic approach to address hyperkalemia is with the use of a potassium-binding resin (sodium polystyrene sulfonate or calcium polystyrene sulfonate). The resin is added to the liquid feed with active mixing, followed by at least 30 min for the resin to settle, and decanting the liquid feed once the gel has formed. Studies have shown a variable reduction in serum potassium levels in infants fed breastmilk and IF using this approach, but there are also other effects: an increase in serum iron, sodium, sulfur, and pH and a decrease in calcium, copper, manganese, phosphate, and zinc [64–72]. Pre-treatment of IF with these resins has also resulted in hypokalemia, hypernatremia, and hypocalcemia [64]. It is advisable to monitor electrolytes and micronutrients that may be altered by these potassium binders, but they are rarely used for long-term management as they carry a high risk of causing severe constipation and even bowel necrosis. More recently, pre-treatment with patiromer (a calcium-based cation exchange polymer) has been found to decrease the potassium content of IF, with an increase in calcium, magnesium, sodium, and phosphate [73].

Dietary potassium intake increases with the introduction of solid foods, the first foods often being vegetables, potatoes, and fruits, which have a high potassium content that may potentially aggravate hyperkalemia. The routine omission of vegetables and fruits from the diet based simply on their potassium content should be discouraged, considering that the bioavailability of potassium in unprocessed plant foods is no more than 60% [74]. These foods also offer other nutritional benefits such as vitamins, minerals, and fiber. For adults, Kidney Disease Improving Global Outcomes (KDIGO) suggests an overall healthy dietary pattern promoting low potassium plant-based foods, especially vegetables [75]. It has also been suggested that it may be beneficial to choose foods with a low potassium to fiber ratio to enable a higher fiber intake to be maintained while lowering dietary potassium [76]. There is no reason why infants should not benefit from a diet rich in fruits, vegetables, and whole grains.

Fruits and vegetables with a lower potassium content can be offered (Table 8), along with advice on portion size of other high nutritional value foods with a high potassium content: meat, poultry, fish, eggs, pulses (peas, beans, lentils) and cereals (grains). The potassium content of refined cereals is lower than whole grain varieties, but the bioavailability of potassium may be higher; they are also lower in other essential nutrients and fiber, so they may not be the preferred choice. Families may find it helpful to have lists of foods with low, moderate, and high potassium content with a daily allowance of each category. However, it is not usually necessary to offer this as the infant’s appetite is generally low and the small amounts of foods taken are of no concern.

Cooking methods can reduce the high potassium content of potatoes and other tuberous roots and legumes; cooking in ample water reduces potassium content by 35–80% while soaking raw food has very little effect [70, 77–88]. Cutting potatoes into small pieces and then double-cooking (bringing the water to a boil and then replacing it with fresh water) reduces the potassium content further [81, 82]. Microwave cooking reduces the potassium content of foods, but to a lesser extent than boiling [87]. Sous-vide cooking [89] and frying [85] increase potassium content of foods. Caregivers should be advised about these cooking methods by a dietitian, as boiling also reduces the amounts of other minerals and water-soluble vitamins.

Once the infant is taking a mixed diet, the use of renal-specific low potassium IF instead of whey-dominant IF allows for the inclusion and greater variety of high potassium foods. Processed foods containing potassium additives should be avoided as they provide an unnecessary source of potassium with high (90–100%) bioavailability [90, 91].

In the case of hypokalemia, the initial approach is to address any medical causes, such as excessive dialysate potassium losses, medications (potassium binding resins, diuretics), gastrointestinal losses (vomiting, diarrhea), or metabolic alkalosis. Severe, life-threatening hypokalemia requires prompt medical intervention, usually with intravenous potassium infusion. If there is persistent hypokalemia, dietary potassium intake should be increased by including higher potassium-containing foods. If a low potassium IF formula is being used, this should be changed to a whey-dominant IF in a stepwise fashion.

### Sodium

Polyuric infants with CKD have excessive urinary salt losses and may require sodium supplements (sodium chloride or sodium with a base if metabolic acidosis is also present) to replace these losses. When complementary solid foods are introduced, these infants often prefer salty tastes. Alternatively, infants with CKD4–5D may have sodium and fluid retention, which can result in volume overload, edema and hypertension. Breastmilk and whey-dominant IF should continue as the source of nutrition for the young infant as they are low in sodium (around 15 mg/100 mL) content. For the older infant, home-prepared foods are usually low in sodium, provided no salt is added, and salty foods, e.g., cheese and processed meats and fish, should be avoided. The content of commercial weaning foods is regulated, and they are low in salt.

## Key summary points

- Breastmilk or whey-dominant infant formula are the preferred feeds for infants with CKD.
- Infants are at risk for growth failure. Measure euvolemic (dry) weight, length and head circumference at frequent intervals and plot on growth charts.
- Commence nutritional supplementation as soon as weight begins to falter. Poor nutritional intake in the first year of life may lead to irreversible loss of final adult height potential.
- Prepare complementary solids from freshly-cooked foods to avoid potassium and phosphate additives, and salt, which are commonly added to processed foods. Offer a healthy balanced diet with a wide range of food choices, tastes and textures to promote the development of oral motor skills and reduce the risk of feeding problems.
- Dietary modifications can help maintain serum levels of potassium, calcium and phosphate within age-appropriate normal ranges. Dietary intervention should be based on serial serum measurements.
- Initiate enteral tube feeding when the infant struggles to take sufficient nutrition by mouth.
- Ideally, infants with CKD are best cared for in a center with a pediatric renal dietitian who works closely with physicians to ensure optimal nutritional management.

## Multiple choice questions

1. Which of the following statements is true?
  - The infant’s weight, length and weight-for-length need only be measured when there are concerns about growth.
  - The infant’s euvolemic weight, length and head circumference should be measured frequently and plotted on WHO growth charts.
  - True gain in body weight in infants on peritoneal dialysis cannot be distinguished from fluid retention.
  - An infant’s failure to gain weight is not a signal for nutritional intervention.
2. Which of the following statements is true?
  - The energy and protein intake for the conservatively managed infant with CKD should approximate that of the healthy infant.
  - The energy and protein intake for the conservatively managed infant with CKD should be 150% higher than the requirements of the healthy infant.
  - Infants with CKD on peritoneal dialysis require a lower protein intake than infants managed conservatively.
  - The energy derived from glucose in the dialysate should not be included in the energy intake for the infant on peritoneal dialysis.
3. Which of the following statements is true?
  - Infants with CKD should be fed a renal-specific low potassium formula from birth.
  - Infants with CKD can have cow’s milk as their main drink from 6 months of age.
  - Breast milk or whey-dominant infant formula are the preferred feeds for infants with CKD throughout the first year.
  - Casein-dominant infant formulas have a nutritional profile closest to breast milk.
4. Which of the following statements is true?
  - Infants with CKD should have a healthy balanced diet avoiding sugars, fats and salt.
  - Infants with CKD should avoid foods high in calcium, phosphate and potassium.
  - Modification of the calcium, phosphate and potassium content of the diet of the infant with CKD can help maintain normal serum ranges.
  - The introduction of solid foods should be delayed in the infant with CKD.
5. Which of the following statements is true?
  - As soon as the infant’s weight starts to falter, start enteral tube feeding.
  - As soon as the infant’s weight starts to falter restrict the intake of phosphate and potassium.
  - As soon as the infant’s weight starts to falter, start peritoneal dialysis.
  - As soon as the infant’s weight starts to falter, start nutritional supplementation of oral feeds and solid foods.

## Declarations

### Disclaimer

The Pediatric Renal Nutrition Taskforce wishes to confirm that Vitaflo has not influenced the content of this Educational Review.
